# Cellular Senescence in Brain Aging

**DOI:** 10.3389/fnagi.2021.646924

**Published:** 2021-02-25

**Authors:** Ewa Sikora, Anna Bielak-Zmijewska, Magdalena Dudkowska, Adam Krzystyniak, Grazyna Mosieniak, Malgorzata Wesierska, Jakub Wlodarczyk

**Affiliations:** ^1^Laboratory of Molecular Bases of Aging, Nencki Institute of Experimental Biology, PAS, Warsaw, Poland; ^2^Laboratory of Neuropsychology, Nencki Institute of Experimental Biology, PAS, Warsaw, Poland; ^3^Laboratory of Cell Biophysics, Nencki Institute of Experimental Biology, PAS, Warsaw, Poland

**Keywords:** autophagy, brain aging, cellular senescence, cognitive impairment, neuroinflammation, neuronal plasticity

## Abstract

Aging of the brain can manifest itself as a memory and cognitive decline, which has been shown to frequently coincide with changes in the structural plasticity of dendritic spines. Decreased number and maturity of spines in aged animals and humans, together with changes in synaptic transmission, may reflect aberrant neuronal plasticity directly associated with impaired brain functions. In extreme, a neurodegenerative disease, which completely devastates the basic functions of the brain, may develop. While cellular senescence in peripheral tissues has recently been linked to aging and a number of aging-related disorders, its involvement in brain aging is just beginning to be explored. However, accumulated evidence suggests that cell senescence may play a role in the aging of the brain, as it has been documented in other organs. Senescent cells stop dividing and shift their activity to strengthen the secretory function, which leads to the acquisition of the so called senescence-associated secretory phenotype (SASP). Senescent cells have also other characteristics, such as altered morphology and proteostasis, decreased propensity to undergo apoptosis, autophagy impairment, accumulation of lipid droplets, increased activity of senescence-associated-β-galactosidase (SA-β-gal), and epigenetic alterations, including DNA methylation, chromatin remodeling, and histone post-translational modifications that, in consequence, result in altered gene expression. Proliferation-competent glial cells can undergo senescence both *in vitro* and *in vivo*, and they likely participate in neuroinflammation, which is characteristic for the aging brain. However, apart from proliferation-competent glial cells, the brain consists of post-mitotic neurons. Interestingly, it has emerged recently, that non-proliferating neuronal cells present in the brain or cultivated *in vitro* can also have some hallmarks, including SASP, typical for senescent cells that ceased to divide. It has been documented that so called senolytics, which by definition, eliminate senescent cells, can improve cognitive ability in mice models. In this review, we ask questions about the role of senescent brain cells in brain plasticity and cognitive functions impairments and how senolytics can improve them. We will discuss whether neuronal plasticity, defined as morphological and functional changes at the level of neurons and dendritic spines, can be the hallmark of neuronal senescence susceptible to the effects of senolytics.

## Introduction

As with other organs and systems, the functional capabilities of the brain decline progressively during aging. As we age, cognitive performance generally declines which manifests as decrements in learning and memory, attention, decision-making speed, sensory perception (vision, hearing, touch, smell, and taste), and motor coordination (reviewed in Mattson and Arumugam, 2018). Aging is the leading risk factor of age-related diseases (ARDs), including neurodegenerative disorders. The aging process and ARDs are considered as a sort of a continuum with two extremes. One is represented by centenarians, who largely avoided or postponed most ARDs and are characterized by decelerated aging. Individuals 60+, 70+, 80+ who suffered from one or more severe ARDs, represent another extremum and show signs of accelerated aging. In between, there are relatively healthy aged people (Franceschi et al., 2018). Thus, precise boundaries between “normal” and “pathological” aging do not exist, especially when molecular and cellular mechanisms at the roots of aging are considered. Particularly, little is known about healthy brain aging outside of the realm of neurogenerative diseases, such as Alzheimer's disease (AD) and Parkinson's disease (PD). Nonetheless, we must remember that the current consensus in geroscience (Kennedy et al., 2014) considers AD as a more severe form of pathologies associated with normal aging. With age physical fitness and cognitive functions often decline in human and animals (Leal and Yassa, 2015). However, we must not forget that in nature exist animal species, such as naked mole rats, ocean quahog, rockfish and Greenland shark, and many others that exhibit negligible senescence and superior resistance to age-related diseases (Finch, 2009).

In humans, cognitive abilities can be divided into such domains as: processing speed, attention, memory, language, visuospatial abilities, and executive functioning (Harada et al., 2013). Similarly, animal behavior is based on attention and different kinds of memory. Age-associated deterioration of cognitive functions correlates with impaired motor coordination of both animals and humans, who lose their independence and experience a decrease in the quality of life. The age-related cognitive impairment is associated with changes in the central nervous system, mainly in the prefrontal cortex and hippocampus. These changes may lead to development of not only neurodegenerative diseases, but also psychiatric diseases, for example, depression and schizophrenias (Baker and Petersen, 2018). However, in agreement with the idea of a continuum of the aging process (Franceschi et al., 2018), healthy aging, free of mental disabilities, is not a rare exception.

Transcriptional profiling of the human frontal cortex from individuals ranging from 26 to 106 years of age defines a set of genes with reduced expression after the age of 40. Genes that play a role in synaptic function and neuronal plasticity that underlies learning and memory, were among those most significantly affected in the aging human cortex. Significantly reduced expression of several neurotransmitter receptors that play a key role in synaptic plasticity, including the GluR1 AMPA (a-amino-3-hydroxy-5-methyl-4-isoxazole propionic acid) receptor subunit, the NMDA (Nmethyl- D-aspartate) R2A receptor subunit, and subunits of the GABA receptor, was shown in people over 40. Moreover, the expression of genes that mediate synaptic vesicle release and recycling, involved in protein transport, involved in protein turnover, also showed reduced expression in the aged cortex. Interestingly, most of the age-downregulated genes showed significantly greater oxidative DNA damage in the aged cortex. In line with this, the aging of the human frontal cortex was associated with increased expression of genes that mediate stress responses and repair. Those included genes involved in protein folding, DNA damage repair, antioxidant defense, metal ion homeostasis, and neuroinflammation (Lu et al., 2004). Generally, the study of Lu et al. may suggest that the main culprit of aging could be cellular senescence of brain cells.

Actually, the hallmarks of aging that are common to neurons and other cells have been described recently (Mattson and Arumugam, 2018). They include: mitochondrial dysfunction, intracellular accumulation of oxidatively damaged proteins, nucleic acids, and lipids, dysregulated energy metabolism, impaired cellular “waste disposal” mechanisms (autophagy-lysosome and proteasome functionality), impaired adaptive stress response signaling, compromised DNA repair, dysregulated neuronal Ca^2+^ homeostasis, stem cell exhaustion, and inflammation. Since the brain function relies on the neuronal network connectivity, the effects of aging are manifested at the level of synaptic plasticity, as shown by the age-associated decline in the number of synapses and in aberrant synaptic transmission in several brain regions. Moreover, a landmark of the aged brain is an increased level of neuroinflammation generated by glial cells, which can contribute to alterations in neuronal/synaptic function (Lupo et al., 2019).

**Figure 1:**
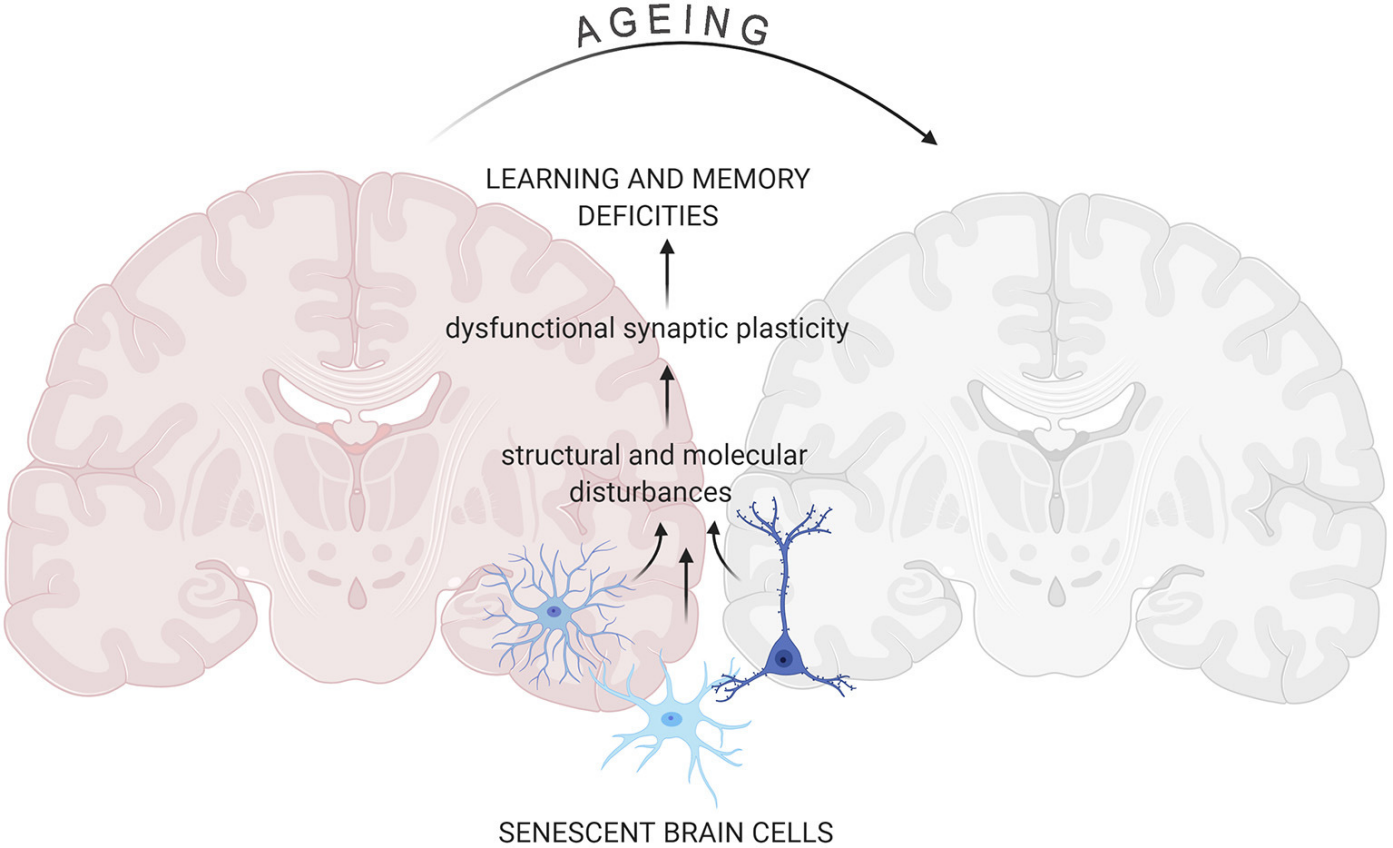
Senescent cells impact on aging-related changes in the brain. Accumulation of senescent glia cells and neurons lead to structural and functional changes in the brain that result in cognitive impairment. All figures were prepared using BioRender.com.

Very recently an outstanding progress in elucidating molecular changes associated with cognitive decline through genome-wide profiling of aging brain cells at different molecular levels, namely genomic, epigenomic, transcriptomic, and proteomic, has been made (Ximerakis et al., 2019). Although the research of the role of cellular senescence in the aging brain is still in its infancy the concept has been laid (Tan et al., 2014; Baker and Petersen, 2018; Kritsilis et al., 2018; Wengerodt et al., 2019) (Figure 1). Therefore, in these review, we will focus on cellular senescence of the brain and discuss recent studies, which have shown that elimination of senescent cells can lead to alleviation of brain associated age-related diseases in many genetically modified mouse models (reviewed by Sikora et al., 2019). Accordingly, we ask the question of whether elimination of senescent brain cells may lead to brain rejuvenation.

## Synaptic Plasticity

Information processing in the brain is a highly complex process; however, it relies on the activity of neurons interconnected at synapses. Strength and efficiency of those neuronal network connections change in response to environmental stimulation enabling the brain to maintain the information, process it and initiate the appropriate response. Modification of synaptic transmission is called synaptic plasticity and could manifest as morphological, electrophysiological changes as well as changes in synaptic protein content.

The number of functional connections in the brain translates into the ability of the neuronal network to store and process information. Quantitative and qualitative measurement of neuronal morphology at the level of dendrite arborisation and morphology of dendritic spines that host excitatory synapses is a tool widely used to assess synaptic plasticity. Hippocampus together with Prefrontal Cortex (PFC) are brain regions critical for cognitive abilities that have been extensively studied in the context of aging-associated changes. Most of the excitatory synapses are located on spines placed all along the dendrites. Dendritic complexity and length is critically important for the regulation of neuronal function. In humans, hippocampal neurons appear to retain the size and complexity of their dendritic arborisation throughout life (Flood, 1993). However, there are also studies suggesting that hippocampal dendritic trees of some subregions, such as CA1, could actually extend with age (Turner and Deupree, 1991). In contrast, in cortical neurons of animal and humans, numerous studies showed regression of dendritic arbors in cortical neurons with age. Total dendritic length and complexity decrease with age for apical and basal dendrites (de Brabander et al., 1998). Animal models of accelerated aging partially replicate the changes observed in aged animals and humans. Thus, SAMP8 mice, with accelerating aging exhibit thinner and 45% shorter apical dendrites in medial PFC (Shimada et al., 2006).

Both the density and morphology of dendritic spines may undergo age-associated changes. Similarly to dendritic arborisation, the number of dendritic spines on hippocampal neurons is more stable than on cortical ones. Spine numbers in rat and human CA1 hippocampal region generally stay unchanged with age (Dickstein et al., 2013), whereas regional specific decrease in dendritic spine densities has been reported in CA3 (Adams et al., 2010) and subiculum (Uemura, 1985). A decrease in dendritic spine densities in cortical neurons of aged animals or humans, compared to young controls, has been reported and ranged from 23 to 55% depending on species, cortical region and age (Shimada et al., 2006).

**Figure 2:**
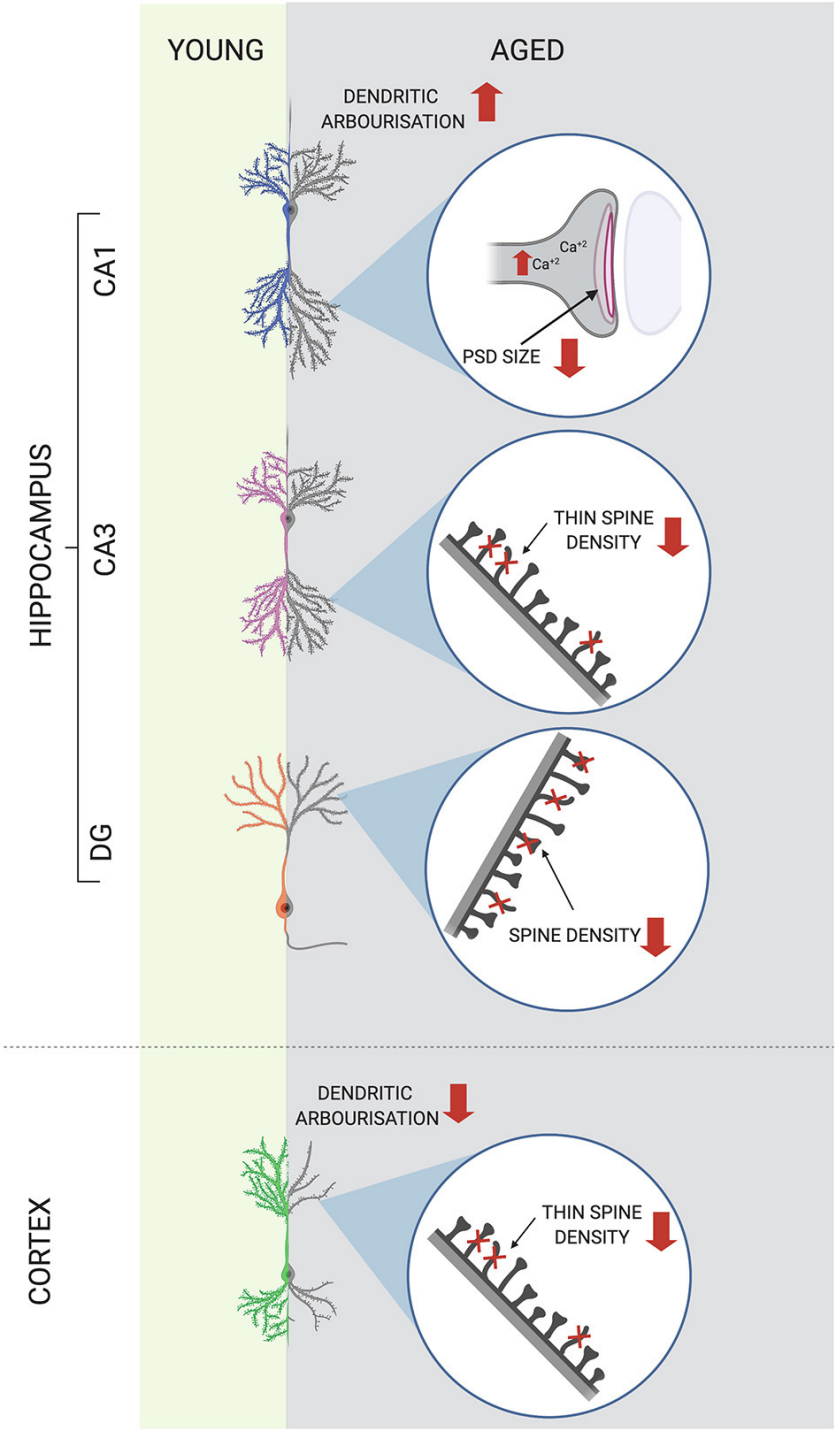
Age-associated changes in synaptic plasticity at hippocampal and cortical neurons. Synaptic plasticity is altered in the aged brain in a region specific manner. Decreased size of postsynaptic density (PSD) of CA1 neurons together with increased baseline level of intracellular calcium ions [Ca^+2^] have been reported in the hippocampus of aged animals. In other hippocampal regions spine densities decrease with age, with selective pruning of less mature spines in CA3. In cortical neurons not only spine densities but also dendritic tree length and complexity decrease with age. **↑**, increase; ↓, decrease.

The picture is even more complex when the morphological variability of dendritic protrusions is taken into consideration. Though spines display a wide morphological continuum, there are four main classes of spine shapes: thin, filopodia (long), stubby (short and wide), and mushroom (large head with thin neck), which represent a different stage of maturation and stability, with thin being the least stable. Other classification is based on the structure of spine postsynaptic density (PSD) of excitatory synapses. PSD is an element of the postsynaptic membrane visible in the electron microscope as a thick plate consisting of densely located receptors for neurotransmitters. Large spines have large heads and often are described as mushroom-like. Those may have PSDs with distinct breaks that are called perforated. Thin spines with small heads typically have small, uniform, non-perforated PSDs (Petralia et al., 2014). In cortical regions, the least stable spines (non-perforated population) have been shown to be most vulnerable to aging-associated decrease whereas the number of stable mushroom spines remained unaltered (Bloss et al., 2011). Aging selectively alters the number and function of hippocampal synapses in a region specific manner. CA3 cells of aged animals and humans are characterized by a decreased density of spines. This pruning with aging is selective toward less mature axo-spinous synapses (Adams et al., 2010). In the CA1 region, the number spines is not altered but mature perforated ones display reduction in the PSD area in aged learning-impaired rats (Nicholson et al., 2004). Dendrites, and particularly dendritic spines, are very dynamic structures. Maintenance of the brain neuroarchitecture represents, in fact, a certain balance between sprouting and retraction of membrane protrusions of different brain cells. Therefore, the above-mentioned results could suggest that aged cortical and hippocampal neuronal networks at CA3 region are less plastic, with a decreased ability to create new intraneuronal connections, whereas hippocampal CA1 neurons are more prone to deficits in the most stable synapses associated with cognitive abilities. Age-associated changes in synaptic plasticity at hippocampal and cortical neurons are illustrated on Figure 2.

Electrophysiological studies have confirmed anatomical findings of changes in structural plasticity of the hippocampus. Reduced amplitude of Excitatory Postsynaptic Potential (EPSP) of the hippocampal Schaffer collaterals CA1 synapses, without alternations of unitary EPSP and amplitude of Schaffer Collaterals, points to the loss of functional synapses in the CA1 area (Barnes et al., 1992, 2000). Similarly, decreased synapse density observed in DG has been confirmed by decreased EPSP amplitude, accompanied by lower amplitude of fiber potential at the perforant path–dentate gyrus granule cell synapse. Interestingly, in aged group unitary EPSPs were found to be of higher amplitude in dentate gyrus which shows that compensatory mechanisms are present that would counteract synapse loss by increasing synaptic transmission (Barnes and McNaughton, 1980). Once the classical synapse is formed, signals from one neuron to another can be transferred via neurotransmitter release leading to alteration of transmembrane electrical potential and an action potential in the postsynaptic neuron. Pre- and postsynaptic firing of action potentials can strengthen (long term potentiation, LTP) or weaken (long term depression, LTD) signal transmission depending on patterns of activity (Glasgow et al., 2019). Electrophysiological measurements in acute slices revealed that LTP induction in CA1 cells is impaired only if a weaker stimulus is used. In the case of stronger stimulation (robust high-frequency, high current amplitude stimulation protocol), LTP in the hippocampus of old animals is evoked to a similar extent as that measured in young animals, which suggests a higher threshold for entering LTP induction phase in aged CA1 cells (Landfield et al., 1978). This phenomenon could be explained by dysregulation of Ca^2+^ homeostasis resulting from up-regulation of synaptic L-type Ca^2+^ channels in aged neurons. Elevated intracellular Ca^2+^ causes higher amplitude of slow after hyperpolarization (AHP) by activating calcium driven potassium channels, which reduces cell depolarization in response to stimulation. This, in the long run, may result in an increased probability of LTD induction and in impairments in the maintenance phase of LTP (Thibault and Landfield, 1996). In contrast, CA3 neurons are characterized by an age-related increase in intrinsic excitability and *in vivo* firing rate. In line with findings for CA1, in CA3 pyramidal neurons no upregulation of L-type Ca^2+^ channels has been reported (Maglione et al., 2019) what corroborates with lack of increase in AHP. Increased frequency of action potentials in CA3 pyramidal neurons has been, however, associated with an increase in the fast AHP that was, at least partially, attributed to increased perisomatic expression of A-type K^+^ channels (Simkin et al., 2015). Contrasting effects of aging on different hippocampal sub-regions suggest disruption of optimal CA3-CA1 interactions and subsequent attenuation of oscillatory activity necessary for learning.

Increases in slow AHP have also been observed in cortical neurons; however, they were accompanied by a higher frequency of action potentials (APs) (Chang et al., 2005). In aged monkeys, behavioral performance was dependent on the mean firing rate, which has an optimum. Both decrease and increase in the frequency of APs have been negatively correlated with good performance in cognitive behavioral tasks (Chang et al., 2005).

Summing up, aging affects neuronal plasticity at different levels, from changes in cell morphology through biochemical to biophysical alterations. Despite the fact that these changes are multidirectional and depend on brain region and cell compartment, they all contribute to age-related cognitive deficits.

## Cellular Senescence

Cells are the basic building blocks of any multi-cellular organism. They build the tissue structure and ensure the proper functioning of the entire organism through autonomous and non-autonomous activities. The brain requires multiple cell types, including neurons, astrocytes, microglia, oligodendrocytes, and endothelial cells, working in concert to ensure proper functioning of the organism. All known hallmarks of brain aging (Mattson and Arumugam, 2018) have been studied for many years in other tissues and are associated with cell senescence and autophagy disturbances (Lopez-Otin et al., 2013). From a reductionist point of view, we age as our cells senesce (Campisi, 2001). Interestingly, the process of cellular senescence cannot be reduced to a simple loss of cell function and death. Senescent cells are alive, resistant to apoptosis and their metabolism is strictly linked to autophagy regulation (Gewirtz, 2013).

Cellular senescence was initially ascribed only to the phenomenon of cell division, being defined as an irreversible loss of cell population division potential observed *in vitro* concurrent with an increased cell size (Hayflick and Moorhead, 1961). Later, this type of cell senescence i.e., replicative senescence (RS) has been linked with telomere erosion (Harley et al., 1992). Subsequently, the signaling pathways and hallmarks, taken from the realms of replicative senescence, have been adopted to the general characteristics of cellular senescence. Besides replicative senescence, at least several other types of cellular senescence characteristic for proliferation-competent cells can be distinguished, namely stress-induced premature senescence (SIPS), oncogene-induced senescence (OIS), programmed senescence (PS) taking place during embryogenesis, and therapy-induced senescence (TIS) of cancer cells as reviewed in Bielak-Zmijewska et al. (2018). Interestingly, post-mitotic, non-dividing nerve cells also undergo senescence (Jurk et al., 2012; Piechota et al., 2016). In any case, there are common signaling pathways and hallmarks, such as mitochondria and lysosome impairment, chromatin alterations, proteostasis and autophagy disruption, metabolic changes, decreased propensity to undergo apoptosis and increased secretion of many factors, commonly described as senescence-associated secretory phenotype (SASP) (Gorgoulis et al., 2019). SASP components include many cytokines, metalloproteinases and growth factors among others (Coppe et al., 2010). Different stressors can induce cellular senescence and DNA damage response (DDR), although this is not valid in the case of PS. The DDR is characterized by increased accumulation of γH2AX foci (histone H2AX phosphorylated at Ser139) and p53-binding protein 1 (53BP1) in the chromatin and by activation of a kinase cascade involving the serine/ threonine-non-specific kinases ATM and ATR and at a later stage the checkpoint serine/threonine kinases CHK1 and CHK2. This scenario eventually leads to activation of the p53/p21^CIP1/WAF1^ signaling pathway (reviewed in Bielak-Zmijewska et al., 2018; Herranz and Gil, 2018). The DDR associated with replicative senescence is telomere-dependent because it is connected with an overall loss of telomeric length and telomere uncapping. During OIS, DDR occurs independently of telomeric length, but it is still associated with telomeric dysfunction. Interestingly. DNA damage response in the aging brain may be caused by telomere dysfunction, stress-induced DNA damage or both (Jurk et al., 2012). In fact, data suggest that telomeres might be the source of persistent DDR, even without overt telomere shortening in various mouse tissues including brain (Fumagalli et al., 2012; Hewitt et al., 2012). Thus, the involvement of telomeres and telomerase in aging goes well-beyond telomere shortening and DNA damage. This is particularly interesting in the case of non-dividing post-mitotic cells, what has been highlighted recently (Jacome Burbano and Gilson, 2020; Panczyszyn et al., 2020).

For cycling cells, the increase in the levels cell cycle inhibitors (cyclin-dependent kinases- cdks inhibitors), p21^CIP1/WAF1^ and p16^INK4a^, is the most characteristic feature of senescence (Beausejour et al., 2003). The most useful way of screening senescent cells is to observe their morphology. When senescence is triggered, the cells increase in size and granularity and the senescence-associated- β- galactosidase (SA-β-gal), a lysosomal enzyme, becomes more active (Dimri et al., 1995). Although SA-β-gal activity is not a fully specific marker of cell senescence, it is characteristic for all types of senescence and is easily detected by colorimetric method. It can be assumed that proliferating brain cells can undergo the process of cell senescence, like many other types of cells. Moreover, accumulation of senescent cells have been observed in age and they are the culprit of many age-related diseases (van Deursen, 2014). An interesting issue just emerging concerns senescence of post-mitotic cells, including neurons and their role in aging (von Zglinicki et al., 2021). Accordingly, in the following chapters we briefly describe the so far collected knowledge of brain cell senescence. We are aware, however, that our article does not exhaust all the issues related to this topic.

## Senescence of Glial Cells

Glial cells constitute around 50% of the total amount of cells in the brain and play key roles in regulating brain homeostasis in health and disease. Their essential functions include providing nutritional support to neurons, activation of immune responses, and regulation of synaptic transmission and plasticity (Salas et al., 2020). In contrast to neural cells, glial cells are proliferation-competent, and as such, are prone to undergo canonical cell senescence. Interestingly, a transcriptional study of aging-related changes in gene expression across human brain regions from 480 individuals ranging in age from 16 to 106 years, showed that glial cells, not neurons, displayed the majority of differential gene expression with aging (Soreq et al., 2017).

### Astrocyte Senescence

Astrocytes comprise about 20% of cells in the brain and play an important role in brain physiology and neuronal function. They provide support for neuronal cells, regulate the content of the synaptic cleft, play an essential role in the maintenance of ion homeostasis (e.g., by shunting potassium ions from the regions of high to the regions of low neuronal activity), and maintain blood-brain barrier (BBB) integrity. Acting as a part of the tripartite synapse, they control the concentration of glutamate (Glu), and thus glutamatergic transmission, and secrete their own glial transmitters. They also play a significant role in the recovery after brain injury by reducing wound-derived excitotoxicity, limiting damage, participating in scar formation and, at later post-injury stages, by in ensuring regeneration. Astrocytes also have several immune functions, i.e., they contain several pattern-recognition receptors, and can secrete cytokines and chemokines. In response to an acute injury, there is an increase in reactive astrocytes: cells undergo various alterations including swelling, hypertrophy, proliferation, and increased expression of a cytoskeletal protein, GFAP (Verkhratsky et al., 2010). Interestingly, the increase in the expression of GFAP, the major intermediate filament protein, which is also a specific marker of astrocytes, is the most common change observed in astrocytes with aging. Another intermediate filament, vimentin, which was for a long time considered to be present only in newly-generated and immature astrocytes, also increases with age (reviewed by Salminen et al., 2011). Transcriptomics analysis revealed dysregulation of genes associated with the cytoskeleton, proliferation, immune response, apoptosis, and ubiquitin-mediated proteolysis that occurs in the aging brain (Simpson et al., 2011). Moreover, aging astrocytes may lose their capacity to regulate glutamate level during synaptic activity due to a reduction in the level of astrocyte-specific glutamate transporters. They may also lose their neuronal support and reduce interactions with synapses. The outcome would be altered synaptic function and plasticity, increased neuronal oxidative and proteolytic stress, and mitochondrial dysfunction, which in the hippocampus would lead to dysfunction in memory retrieval and consolidation (Ojo et al., 2015).

The very important question is whether astrocytes can undergo senescence similarly to many other proliferation-competent cells. Indeed, astrocytes have been shown to undergo cellular senescence *in vitro*, both replicative and induced by different external stressors (Cohen and Torres, 2019). Below are some examples.

In response to oxidative stress or proteasome inhibitor, murine, and human astrocytes showed reduced proliferation capability and changes in several established markers of senescence, namely, SA-β-gal activity and in the level of p21^WAF1/CIP1^, p53, and p16^INK4A^ proteins (Bitto et al., 2010). Evans et al. (2003) showed that human astrocytes underwent senescence, which was telomere-erosion independent and p53-dependent. The same group documented an increased p16^INK4A^ level in the frontal cortex area of old and AD people in comparison with young subjects. It was also shown that the senescence process could be triggered in astrocytes by amyloid-β, which induced activation of SA-β-gal, p16^INK4A^ expression and secretory phenotype connected with p38 MAP kinase (Bhat et al., 2012). Others showed that ammonia induced senescence in cultured astrocytes in a glutamine synthesis- and oxidative stress-dependent manner through p53-dependent transcription of cell cycle regulatory genes, p21^WAF1/CIP1^ and GADD45a. Increased p21^WAF1/CIP1^, p53, and GADD45a mRNA expression levels were also found in postmortem brain samples from patients with liver cirrhosis and hepatic encelophaty (Gorg et al., 2015). In another studies, carried out on long-term rat astrocyte cultures, the generation of reactive oxygen species was enhanced and mitochondrial activity decreased. Simultaneously, there was an increase in proteins that stained positively for nitrotyrosine. The expression of Cu/Zn-superoxide dismutase (SOD-1), heme oxygenase-1 (HO-1), and inducible nitric oxide synthase (iNOS) as well as glutamate uptake were increased in aged astrocytes. On the 90th day *in vitro* the cells were SA-β-gal-positive and had increased expression of GFAP (Pertusa et al., 2007). Recently, it has been documented that treatment of astrocytes with sirtuins's inhibitor, tenovin-1 (Bang et al., 2019b), or etoposide (Bang et al., 2019a), increased SA-β-gal-positive cell number, induced the senescence-associated secretory phenotype, including increased expression of IL-6 and IL-1β, and of cell cycle-related proteins, such as phospho-histone H3 and CDK2 (Bang et al., 2019a). Others showed that in human astrocytes induced to senesce by X-irradiation, there was downregulation of genes encoding glutamate and potassium transporters. This downregulation led to neuronal cell death in co-culture assays, suggesting a pivotal role of cellular senescence, particularly in astrocytes, in excitotoxicity, which may lead to neurodegeneration and pathologies such as Alzheimer's disease and related dementias (Limbad et al., 2020). Subsequent findings confirmed that ovarian estradiol induces senescence of hypothalamic astrocytes and that the senescent astrocytes compromise the regulation of progesterone synthesis and GnRH secretion, which may contribute to the aging-related decline in female reproductive function (Dai et al., 2020). HIV and methamphetamine were shown to induce astrocyte senescence in *in vitro* culture and several animal models (Yu et al., 2017).

### Microglia Senescence

Microglia, which make up several percent of all brain cells in the healthy young CNS, have a typical ramified morphology and are distributed throughout the neural parenchyma and cover all regions of the CNS (Wong, 2013).

During aging, microglia undergo substantial changes in distribution, morphology, and behavior (Spittau, 2017). These include: increase in the number/density, decrease in regularity in distribution, reduction of individual ramification (dendritic arbor area, branching, and total process length), appearance of morphological changes suggestive of increased activation state (e.g., perinuclear cytoplasmic hypertrophy, retraction of processes), appearance of dystrophic microglia in aged human brains, decrease in the rate of process movement, and decrease in the rate of migration to focal tissue injury (Wong, 2013). Dystrophic microglia were shown to possess increased sustained inflammatory response in reaction to damage (Damani et al., 2011). Importantly, microglial dystrophy is widespread in advanced AD as well as in Down syndrome and is frequently colocalized with neurofibrillary degeneration (Streit et al., 2020).

Aged microglia are found to express increased levels of effector molecules associated with activated microglia. Increased expression of inflammatory cytokines, such as IL-1β, TNF-α, IL-6, was detected in aged microglia studied *in situ*, isolated *ex vivo* or cultured *in vitro* (reviewed by Wong, 2013). Due to telomere erosion observed in cultured microglia (Flanary and Streit, 2004) and associated with dementia in human AD brain samples (Flanary et al., 2007), and to lipofuscin accumulation and mitochondria dysregulation, manifested as increased ROS production, it was suggested that microglia underwent cell senescence. However, only recent studies, based on the flow cytometry analysis, revealed an increase in the number of p16^INK4a^ and p21^WAF1/CIP^ positive microglia derived from old mouse brain. This increase correlated with upregulation of autofluorescence (characteristic for senescent cells due to the lipofuscin accumulation), and γH2AX and Bcl-2 (anti-apoptotic protein) level in those cells (Ritzel et al., 2019).

## Neuronal Senescence

Once mature, most of our neurons last our entire lifetime and show high potential for plasticity, which allows them to continuously modulate and adapt their complex synaptic network to changing conditions. The concept of neuronal senescence is relatively new even though it is well-established that neuronal functions decline with aging (Tan et al., 2014). As the hallmarks of cell senescence encompass more than just irreversible growth arrest, the studies to identify markers of neuronal senescence, that is senescence of proliferation-incompetent cells, were recently performed both *in vivo* and *in vitro* (Tan et al., 2014; Kritsilis et al., 2018; Wengerodt et al., 2019). However, data concerning this subject are still scarce, probably due to the existing paradigm telling that cellular senescence is an irreversible/permanent cell cycle arrest. It seems that depending on the type of cell senescence, this arrest can occur either in the G1 or G2 phase of the cell cycle (Bielak-Zmijewska et al., 2014). Since neurons are post-mitotic, non-cycling cells (permanently in the G0 phase of the cell cycle), neuronal senescence, like that of other post-mitotic cells, must rely on other foundations than proliferation arrest. Some markers of cellular senescence were observed in long-lasting neuronal cultures without additional inducer. Generally, it takes 30–40 DIV (days *in vitro*) to develop a senescence phenotype. Although not fully specific, the most useful marker of cell senescence is the increased activity of SA-β-gal. Indeed, increased SA-β-gal was observed in the hippocampus of aging rats (Geng et al., 2010) and long-term *in vitro* cultures of hippocampal (Dong et al., 2011) and cerebellar granule neurons (Bhanu et al., 2010). We also observed stronger staining for hippocampal SA-β-gal in old than young mice. Moreover, in long-term neuroglial co-cultures (neural plus glial cells) of rat cortical cells, we observed a quite rapid increase in SA-β-gal-positive neurons and increased IL-6 production but, interestingly, no hallmarks of DNA damage (Piechota et al., 2016). Moreover, induced DNA damage did not increase the number of SA-β-gal-positive neurons. Similarly, in rat hippocampal neurons cultured *in vitro*, no increase of γH2AX foci was observed (Ishikawa and Ishikawa, 2020). However, Jurk et al. (2012) documented that Purkinje neurons of the cerebellum, and also cortical, hippocampal, and peripheral neurons in old mice, had severe DNA damage, activated p38 MAP kinase, high ROS production and oxidative damage, high interleukin IL-6 production, heterochromatinization and SA-β-gal activity. Moreover, they showed that these senescence symptoms were dependent on DNA damage-induced expression of p21^WAF1/CIP1^. Others showed some features of cellular senescence, such as increased SA-β-gal activity, oxidative stress, γH2AX expression, IL-6 production and astrogliosis, in mixed rat neuroglial co-cultures (Bigagli et al., 2016). Moreno-Blas et al. showed that in rat mixed cultures, both neuronal and glial cells became SA-β-gal-positive, but only neurons expressed p21^WAF/CIP1^ and only astrocytes accumulated γH2AX foci (Moreno-Blas et al., 2019). Moreover, rat senescent neuronal cells *in vitro* were characterized by elevated SASP. In long-term cultures enriched in hippocampal neurons several cell senescence markers were found: upregulation of Cdk inhibitor p16^INK4A^ (Cdkn2a), but not p21^WAF1/CIP1^ (Cdkn1a), increased activation of p38 MAP kinase and loss of lamin B1, which leads to chromatin changes. Neurons at 28 DIV showed upregulation of SASP gene expression including those encoding Cxcl1, plasminogen activator inhibitor-1 (Pai-1), and insulin-like growth factor-binding proteins (Ishikawa and Ishikawa, 2020). The authors documented that not only hippocampal but also cortical cell culture led to expression of senescence markers. Recently, we have focused our interest on senescence of neural progenitor cells (NPCs) derived from A-T (Ataxia telangiectasia) reprogrammed fibroblasts. We showed that A-T NPCs, obtained through neural differentiation of iPSCs in 5% oxygen, possessed some features of senescence, including increased activity of SA-β-gal, increased secretion of IL-6 and IL-8 and reduced sirtuin 1 level in comparison to control NPCs. This phenotype of A-T NPC was accompanied by elevated oxidative stress. Additional sources of oxidative stress, such as increased oxygen concentration (20%) or H_2_O_2_ treatment, aggravated the phenotype of senescence (Sunderland et al., 2020). Since the ATM kinase is a key protein involved in DNA damage response, our data indicate that senescence of neural cells is not DNA damage-dependent and that rely rather on oxidative stress, similarly to rat cortical cell senescence (Piechota et al., 2016). In A-T, but not in control human neural cells, we observed a REST increase. REST is a transcriptional repressor that is downregulated during neuronal development in humans to de-repress neuron-specific genes. However, in the aged brain, REST becomes upregulated to repress apoptosis-inducing genes, thereby facilitating neuronal cell survival (Lu et al., 2014). Accordingly, we postulated that REST could be a reliable marker of neuronal senescence (Sunderland et al., 2020). Indeed, in line of this, Ishikawa and Ishikawa also showed increased REST level in rat senescent cells *in vitro* (Ishikawa and Ishikawa, 2020). It has been documented that also epigenetic modifications play a crucial role in age-related changes in neurons. Histone loss, increased open chromatin regions, altered histone modifications, and changes in DNA methylation (DNAm) pattern lead to alterations in the gene expression profile of aged neurons and, eventually, to neuronal dysfunction. Moreover, impairment of nuclear pores (nuclear pore “leakiness”) contributes to age-related changes in the nucleus and cytoplasm configuration (Schlachetzki et al., 2020). However, the brain is composed of thousands of different neural cell types, and the effects of aging are likely to vary among different brain regions and cell types, perhaps even in opposite directions (Ximerakis et al., 2019).

Collectively, these data showed that, indeed, senescence of post-mitotic nerve cells is a fact, although results published so far are inconsistent due to different culture conditions and measurements. Certainly, more research is needed. It seems that the research on neuronal senescence has gained impetus and we should expect a lot of new data on this topic soon. Moreover, there is a growing body of evidence showing that autophagy, the activity of which is decreased in old animals and humans, is strictly linked to neuronal cell senescence.

## Selected Aspects of Cellular Senescence and Their Role in Brain Aging

Senescent cells display a complex phenotype with multiple effector mechanisms (Gorgoulis et al., 2019). Their strength and combination may differ depending on the senescence trigger and cell type and they influence on the functioning of senescent cell. Among those effector mechanisms epigenetics, autophagy and SASP gained much attention and there is evidence for their critical involvement in the senescence process. Thus, we decided to present the state of the art concerning the role of selected effector mechanism in aging of the brain and senescence of brain cells.

### Epigenetic Changes in Aging Brain and Senescence

One of the most dramatic molecular changes observed in the aging brain, and influenced by lifestyle, is the alteration of the epigenetic mechanisms controlling gene expression. Epigenetic mechanisms regulate a plethora of brain functions including activity-dependent transcription, synaptic plasticity, learning and memory, and adult neurogenesis. In fact, in some brain structures, such as hippocampus and prefrontal cortex, various genes linked to synaptic plasticity and synaptic structure have been shown to be downregulated as a consequence of epigenetic alterations. The epigenetic mechanisms altered in aging include DNA methylation, chromatin remodeling and non-coding RNA expression pattern. Most of these epigenetic alterations contribute to cognitive decline during aging (reviewed by Harman and Martin, 2020). DNA methylation is a process, by which methyl groups are added to cytosine residues, primarily in the regions rich in cytosine-guanine dinucleotides, CpG, which are grouped in so called CpG islands (reviewed in Jiang and Guo, 2020). In neuronal cells, CpG methylation plays a critical role in neural plasticity (Martinowich et al., 2003; Miller et al., 2010).

Human DNA methylation (DNAm) changes, referred to as “epigenetic clocks,” have been widely used to identify differences between chronological age and biological age in health and disease including neurodegeneration and dementia. While in the majority of studies global DNA hypomethylation was observed in various tissues with age, DNA methylation patterns are distinct between tissue and cell types (Mendizabal et al., 2019). It is therefore not surprising that DNAm age estimation models may differ in accuracy across tissue types. Interestingly, a novel epigenetic clock, that performs optimally in human cortex tissue and has the potential to identify phenotypes associated with biological aging in the brain, has been proposed recently. This DNAm clock is specifically designed for the human cortex and is accurate across the whole human lifespan including older donors (aged over 60 years). The authors demonstrate that their clock outperforms the existing DNAm-based predictors developed for other tissues, minimizing the potential for spurious associations with aging phenotypes relevant to the brain (Shireby et al., 2020).

Chromatin remodeling driven by histone modifications is a tightly regulated process whereby modifications (methylation and acetylation of lysine) are added and removed by enzymes, the amount, regulation and function of which are altered during aging (Pal and Tyler, 2016; Gadecka and Bielak-Zmijewska, 2019). The expression of HATs (histone acetyl-transferases) and HDACs (histone deacetylases) and related regulatory molecules has been reported to be altered in the aged brain, and could be linked to age-related alterations in gene transcription (Barter and Foster, 2018).

At the cellular level age-related epigenetic changes can be observed as chromatin rearrangement that leads to dissociation of heterochromatin from the nuclear periphery (Freund et al., 2012). The attachment of heterochromatin to the nuclear membrane is mediated by lamin B1, which preferentially binds histone H3 trimethylated on lysine 27 (H3K27me3) and 9 (H3K9me3) i.e., modifications associated with transcriptional repression (Yang and Sen, 2018). Age-related decline of lamin B1 and a decreased level of H3K27me3 and H3K9me3 entail a diminished heterochromatin content.

Epigenetic changes in chromatin influence SASP gene expression. A good example of a SASP -regulating enzyme is histone deacetylase sirtuin 1 (Sirt1). The level and activity of Sirt1 are going down during aging and cell senescence, which, in turn, provokes an increase in the activity of NF-κB transcription factor, necessary for transactivation of SASP members (Grabowska et al., 2017).

The nuclear periphery, specifically nuclear lamina and the long-lived components of the NPC (nuclear protein complexes) responsible for the communication between nucleus and cytoplasm, are prime targets of cellular senescence and it can be anticipated that they also operate in senescing brain cells (Moreno-Blas et al., 2019; Schlachetzki et al., 2020), however, this statements needs definitely more experimental studies.

### Autophagy in Aging Brain and Brain Cells

Autophagy is an evolutionally conserved intracellular process that enables cells to dispose of damaged or unnecessary molecules and organelles. Thus, autophagy is responsible for cellular homeostasis. Recent genetic evidence indicates that autophagy has a crucial role in the regulation of animal lifespan and that basal level of autophagic activity is essential for longevity (Nakamura and Yoshimori, 2018). Several studies demonstrate that hundreds of proteins become highly insoluble with age, in the absence of evident disease processes (Groh et al., 2017) and that autophagy activity in the brain decreases with age in many experimental models (Loeffler, 2019). One of the most striking morphological changes in neurons during normal aging is the accumulation of lipofuscin aggregates. Lipofuscin is an autofluorescent pigment formed by lipids, metals and misfolded proteins, that is especially abundant in nerve cells, cardiac muscle cells and the skin (Moreno-Garcia et al., 2018). Disturbances in proteostasis, along with accumulation of damaged DNA, dysfunctional mitochondria, and lysosomes, point to the failure of the protein degrading and organelle turnover system in aging. Apart from the proteasome, exclusively destined for protein degradation, autophagy is the biological system for degradation of different kinds of molecules and of whole organelles. Indeed, attenuation of both the proteasomal (Low, 2011) and autophagy-dependent degradation with age has been described (Loeffler, 2019). Down-regulation of expression of the key autophagy genes, such as ATG5, ATG7, and BECN1, was demonstrated during aging of the human brain (Lipinski et al., 2010).

**Figure 3:**
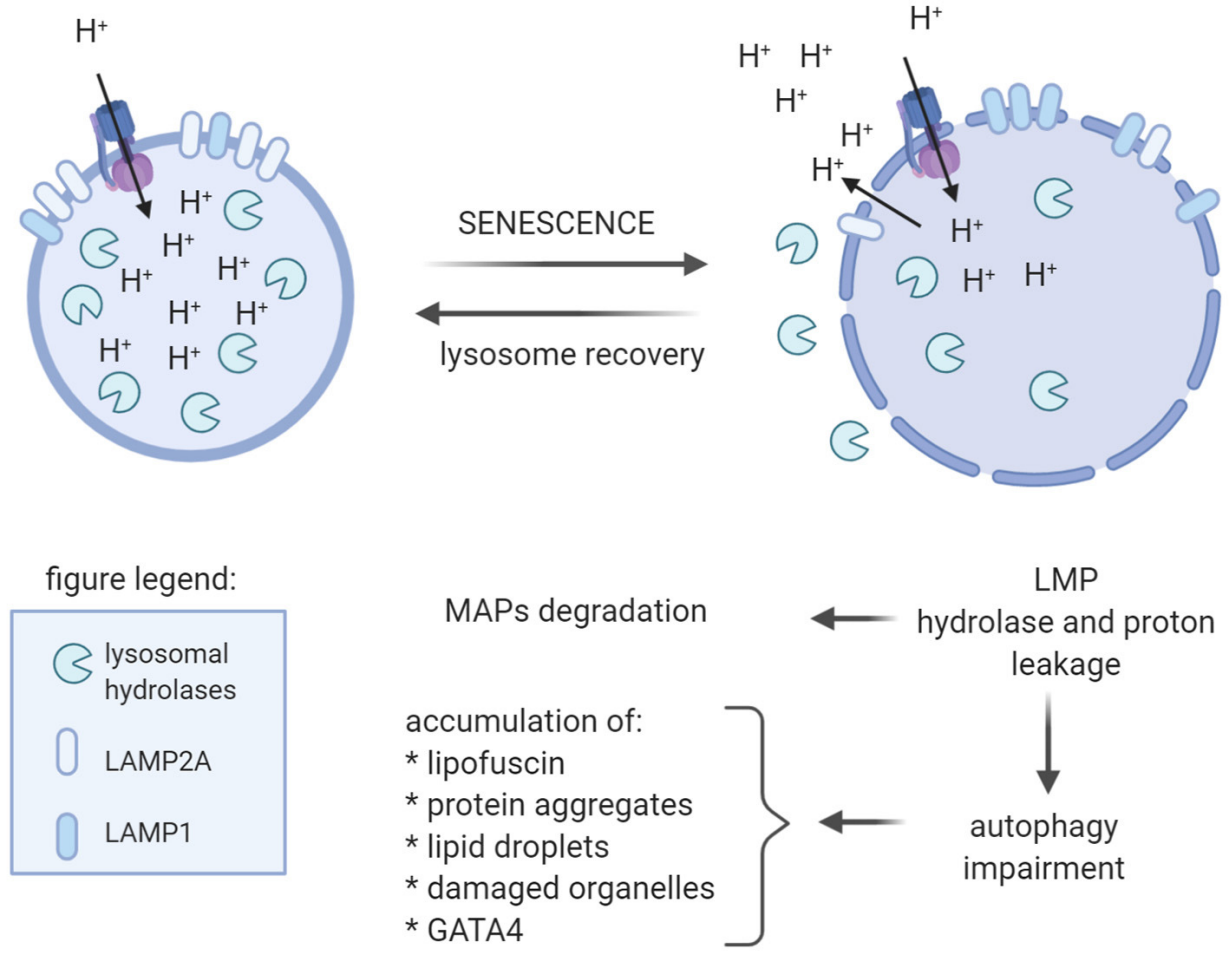
Age-dependent lysosome impairment affects function of the brain cells. With age, lysosomes become large with altered membrane protein content, namely, increased LAMP1 level, at least in quiescent neural stem cells, and decreased level of LAMP2A. Reduced LAMP2A level in lysosomal membrane is believed to be the reason of declined CMA observed in old animals. Additionally, with age, lysosomal membrane permabilization (LMP) occurs resulting in leakage of protons and lysosomal hydrolases into cytosol. Uncontrolled protein hydrolysis, e.g., of microtubule-associated proteins (MAPs), affect their function and may lead to cell death. proton leakage impedes maintenance of low lysosomal pH, which is necessary for proper functioning of hydrolases. Hence, decline in lysosomal function results in autophagy impairment and, in consequence, accumulation of various aggregated proteins, lipofuscin, and damaged organelles.

According to Yim and Mizushima, there are four kinds of autophagy: macroautophagy, chaperon-mediated autophagy (CMA), RN/DNatophagy, and microautophagy (Yim and Mizushima, 2020). However, only during macroautophagy the material destined for degradation is closed in newly formed vesicles called autophagosomes. All autophagy types rely on lysosomes. Thus, their dysfunction due to, for example, lysosomal membrane permeabilization (LMP), leads not only to autophagy impairment but also to leakage of the lysosomal content into cytosol (Figure 3). Hence, damaged lysosomal clearance is important for cell function and survive (Gomez-Sintes et al., 2016). Interestingly, in the aged rat brain, increased cathepsin D activity and microtubule-associated proteins (MAPs), MAP1, and MAP2, degraded by cathepsin D-like enzyme, were found (Matus and Green, 1987; Nakamura et al., 1989). This finding suggested leakage the enzyme from lysosomes. Further investigation demonstrated an increase in other cathepsin in rat brain, as has been summarized by Stoka et al. (2016). Thus, increase in lysosomal enzymes' activity and accumulation of lipofuscin in rat brain points to perturbation of lysosomal function with age.

Indeed, changes in lysosomal activity with age were described by Gomez-Sintes et al. (2016). Aged lysosomes become large and more vulnerable to LMP (Figure 3). It was suggested that changes in lysosomal membrane components resulted in lysosome fragility. Decreased LAMP2A content in lysosomal membrane with age was found in rodents and was postulated to be the reason of age-related decline of CMA (Kiffin et al., 2007). Aged lysosomes were also characterized by increased pH, which affects lysosomal enzyme activity. In consequence, lysosomes accumulate undigested material, including lipofuscin, which in turn, may affect lysosomal function and integrity causing LMP.

Lipidation is the very initial step in autophagy regulated by many autophagic proteins. It means conjugation of the cytosolic LC3-I protein to phosphatidylethanolamine in the phagophore membrane to form LC3-II, which is one of the markers of autophagic vesicles (Yoshii and Mizushima, 2017). Recently, it has been demonstrated that autophagy lipidation machinery has a non-canonical, autophagy-independent function in regulating axonal microtubule dynamics (Negrete-Hurtado et al., 2020). ATG proteins involved in lipid conjugation of LC3 facilitate microtubule-dependent axonal transport. Depletion of these ATG or increase in non-lipidated LC3-I, impaired microtubule dynamics and axonal cargo transport leading to the formation of axonal swelling phenotype. Such changes in the axons are known to compromise learning and memory-dependent neuronal activity that is known to decline with age. Thus, autophagy impairment may promote the accumulation of undigested material but also hampers axonal transport in neurons. The authors suggest that pharmacological activation of autophagy may not only promote the degradation of cytoplasmic material but also impair axonal integrity via altering microtubule stability.

Leeman et al. demonstrated age-related changes in quiescent and activated populations of neural stem cells (NSCs) in the mouse brain (Leeman et al., 2018). Quiescent NSCs were characterized by large lysosomes with high expression of LAMP1 and aggregate accumulation, accompanied by impairment of autophagic degradation (Figure 3). The lysososmal/autophagy activity worsened with age, leading to decline in activation of quiescent NSC in old mice. Increment in lysosomal function, evoked by nutrient deprivation or by activation of lysosome biogenesis, led to decreased aggregate level and a higher rate of quiescent NSC activation, allowing them to regain a more youthful state (Leeman et al., 2018).

Autophagy improvement may counteract or delay the appearance of these unfavorable changes.

The question is: how is autophagy related to cellular senescence?

In a seminal paper, Narita's group documented autophagy activation upon induction of senescence in normal fibroblasts and showed that autophagy inhibition, by depletion of ATG5 or ATG7, attenuated SASP manifestations, namely IL-6 and IL-8 secretion (Young et al., 2009). Subsequently, the same group proposed model, in which autolysosomes, by degrading aged organelles and proteins, generate a high flux of amino acids and other metabolites needed for biosynthesis. In turn, mTORC1 (mTOR Complex 1) promotes the utilization of these metabolites for the synthesis of the SASP components (Narita et al., 2011). Recently, the downregulation of mammalian Sirt1 protein, considering as a “youth protein” (Grabowska et al., 2017) during senescence and *in vivo* aging has been documented to occur via autophagy (Xu et al., 2020). Also our results (Sunderland et al., 2020) show that the level of Sirt1 is lower in senescing fibroblasts from donor suffering from A–T disease (A–T) and reprogrammed to neural progenitors than in non-senescing cells from a healthy donor. However, neither Xu et al. (2020) nor ourselves (Sunderland et al., 2020) provided any information concerning the senescence phase. Hence, Herranz and Gil (2018) actually distinguished three phases of cell senescence: senescence initiation, early and late senescence, with active autophagy restricted to early stage of cell senescence. Thus, it cannot be excluded that active autophagy is inhibited in the late stage of senescence, especially in neurons.

Results of several papers investigating the relationship between autophagy and cell senescence suggest that autophagy is blocked in brain cells with a senescence phenotype. This is in line with the results presented below, showing that brain aging is linked to impaired autophagy. Regarding autophagy and senescence in brain cells, there are some convincing results obtained in *in vitro* culture. Autophagy is a very dynamic process, and if an accumulation of autophagic vesicle is observed, it is rather a sign of autophagy blockage at the stage of autophagosome fusion with lysosomes or cargo degradation in autolysosomes (Yoshii and Mizushima, 2017). Hence, Moreno-Blas et al. (2019) speculated that in senescent cortical neural cells the lysosome—autophagosome fusion was impaired, what resulted in inhibited autophagic flux. Ishikawa and Ishikawa (2020) came to the same conclusion on the basis of research conducted on hippocampal neural cells. Cell treatment with rapamycin led to activation of autophagy by flux unblocking.

Our study performed on neural progenitor cells revealed that senescence in A-T neural cells proceeded hand in hand with disturbances in autophagy (Sunderland et al., 2020). We documented that even though the autophagic vesicle marker (LC3B II) was elevated, there was accumulation of the protein marker of autophagy cargo (p62/SQSTM1), a higher level of internal autophagy inhibitor (Rubicon) and no reaction to chloroquine treatment in A–T NPCs, which points unambiguously to impaired autophagic flux. One especially interesting result was the increased number of LAMP2-positive vesicles in A–T NPCs (Sunderland et al., 2020). This observation possibly links impaired autophagy to increased SA-β-gal activity, which is, in fact, a lysosomal enzyme. SA-β-gal activity has been correlated with lysosomal mass in a publication by Lee et al. (2006) and in our study on rat neurons (Piechota et al., 2016). Both the abundance of lysosomes and loss of functional autophagy can be a result of the same anomaly. A study by Moreno-Blas et al. (2019) demonstrated that the autophagic flux was blocked in senescent neurons *in vitro* possibly due to dysfunction of lysosomes. Another result linking impaired autophagy with the senescence phenotype is the increased level of GATA4 (Moreno-Blas et al., 2019; Sunderland et al., 2020). GATA4 is a transcription factor that is degraded by autophagy in non-senescent cells. In senescent cells GATA4 is stabilized and activates one of the master regulators of SASP, namely, NF-κB (Kang et al., 2015). Judging from the changes in secretion of interleukins in senescent cells, this seems to be the case.

Altogether, the data suggest that age-related lysosomal dysfunction followed by autophagy impairment is strongly interconnected with senescence of brain cells.

### Neuroinflammation and SASP

For a long time brain has been considered as an immune-privileged organ that is equipped with a separate subset of cells, such as microglia, which are responsible for brain-specific immunological response. Similarly to the periphery, age-related changes that appear in the brain has been correlated with increased neuroinflammation (Lynch, 2010). Upregulation of pro-inflammatory cytokines and their modulators that appear in the aging brain coexists with changes in the microglia status, called “primed” microglia. “Primed” microglia are characterized by increased baseline expression of inflammatory markers and mediators, a decreased threshold of activation and a switch to a pro-inflammatory state, and by exaggerated inflammatory response following immune activation (Dilger and Johnson, 2008; Norden et al., 2015; Fonken et al., 2018). Apart from microglia also astrocytes were shown to promote neuroinflammation. Analysis of the differentially expressed genes in mouse brain revealed that aged astrocytes take on a reactive phenotype of neuroinflammatory A1-like reactive astrocytes. In response to lipopolysaccharide (inducer of inflammation) more A1 reactive astrocytes were formed in aged than young mouse brain. It was also demonstrated that cytokines secreted by microglia were responsible for the induction of the A1 phenotype of aged astrocytes (Clarke et al., 2018), emphasizing the role of cell to cell communication in the propagation of neuroinflammation. Of note, contrary to non-reactive astrocytes, reactive A1 astrocytes could not promote neuronal survival, outgrowth, synaptogenesis and phagocytosis, instead, they induced cell death (Liddelow et al., 2017).

During the organismal lifespan, the interplay between the immune and central nervous system impacts on the proper functioning of the brain. There is a constant cross-talk between the peripheral immune system and the brain. This communication is performed *via* soluble factors like cytokines and growth factors, among others, produced by innate and adaptive response cells present in the CNS. Thus, it is not surprising that, as we age, changes in the immune system participate in the neuroinflammation that appears in the brain. Increased level of pro-inflammatory factors in the blood leads to disruption of the blood-brain barrier (Farrall and Wardlaw, 2009; Montagne et al., 2015). Consequently, increased infiltration of immune cells (Togo et al., 2002; Stichel and Luebbert, 2007) and cytokines from the periphery activates microglia and astrocytes. Both types of cells secret pro-inflammatory cytokines like IL-1β, IL-6, and TNFα and, by this means, propagate immunosenescence from the periphery into the brain. Apart from activated cells, also senescent brain cells can support neuroinflammation due to SASP factors. Moreover, senescent cells are also a source of DAMPs (danger-associated molecular patterns, alarmins) that, when secreted outside the cell, facilitate low grade inflammation during aging (reviewed in Franceschi et al., 2017). Importantly, those DAMPs, derived from cellular debris or damaged proteins, might exit cells passively or packed into so called extracellular vesicles (EVs) (Collett et al., 2018; Katsumi et al., 2019; Marcoux et al., 2019). In the case of senescent cells, increased production of EVs may be involved not only in transmission of these specifically selected proteins and nucleic acids (e.g., miRNA), but also serve to “clean” the senescent cell from unwanted, damaged material that accumulates due to lysosome malfunction (Eitan et al., 2016). In this way the pro-inflammatory signal may be spread to neighboring cells and into the systemic milieu.

We have demonstrated that after a long-term *in vitro* culture of rat cortical neurons, the expression of *IL-6* was significantly higher than in cells cultured for only few days (Piechota et al., 2016). Importantly, increased expression of *IL-6* correlated with neuron-specific upregulation of REST protein – a marker of neuronal senescence (Piechota et al., 2016; Ishikawa and Ishikawa, 2020; Sunderland et al., 2020). Accordingly, Moreno-Blas et al. (2019) proved that cortical neurons cultured *in vitro* for 26 days express a higher level of GATA4 transcription factor, which was shown to participate in the regulation of SASP factors expression (Kang et al., 2015). Moreover, senescent cortical cells secret increased amounts of several cytokines and chemokines, among which MCP-1 was the most significantly altered comparing to young cells (Moreno-Blas et al., 2019). Conditioned medium obtained from senescent cortical cells was able to induce premature paracrine senescence of mouse embryonic fibroblasts (MEFs). Thus, the authors proposed that senescent brain cells can also spread the senescence phenotype *via* secreted factors to nearby healthy brain cells such as astrocytes, microglia or endothelial cells (Moreno-Blas et al., 2019). Studies performed by Ishikawa and Ishikawa (2020) confirmed that long-term culture of cortical and hippocampal neurons correlated with increased expression of selected SASP factors. Enhanced immunostaining of one such factor, Cxcl1, was detected specifically in aging neurons. Furthermore, an increased number of IL-6 expressing cortical and Purkinje neurons were shown in the brain of old mice (Jurk et al., 2012).

While senescence of neurons still remains a subject of debate, and its involvement in the regulation of aged brain functioning through SASP factors needs more experimental support, the role of factors secreted by astrocytes in the aging brain is unquestionable. With age, astrocytes become activated, which is a phenomenon induced by a variety of insults and diseases of CNS. One of the common features of activated astrocytes is secretion of a number of cytokines, chemokines and proteases that can have either beneficial or detrimental role on CNS functioning. Importantly, there is a significant overlap between factors secreted by reactive astrocytes and SASP factors; thus, one can speculate, that activated astrocytes may, at least in part, represent a pool of senescent cells (Cohen and Torres, 2019). An increased level of SASP factor mRNA (TNF-α, IL-1β, IL-6, and IL-8) was detected in the hypothalamus of aged mice and in astrocytes induced to senesce by estradiol treatment *in vitro* (Dai et al., 2020). The key transcription factors mediating pro-inflammatory signals in SASP are C/EBP and NF-κB. Of note, these pathways are also fundamental regulators of inflammatory responses in astrocytes (Cardinaux et al., 2000). Accordingly, it was demonstrated that oxidative stress in cultured human astrocytes could activate inflammatory pathways, namely NF-κB, p38MAPK, and JNK pathways, and stimulate the secretion of IL-6 (Lee et al., 2010). Recently, it has been shown that NF-κB also modulates neuronal morphology and function through activation of complement C3 in glia; however, the symptoms of astrocytic senescence were not analyzed (Lian et al., 2015). One of the targets of C/EBP is TGFβ1, which signaling in the brain increases with aging (Doyle et al., 2010) and can inhibit astrocyte proliferation (Lindholm et al., 1992) but also stimulate the expression of GFAP (de Sampaio e Spohr et al., 2002). TGFβ signaling induces expression of MCP-1 also recognized as a SASP factor, in astrocytes. In turn, MCP-1 triggers monocyte recruitment across the BBB, and thus it has a crucial role in inducing chronic inflammation as that is often observed in Alzheimer's disease (Sokolova et al., 2009). Another important inflammatory factor that can induce cellular senescence in the brain is HMGB1 (high mobility group box-1, alarmin) protein (Davalos et al., 2010). Recently it was shown that HMGB1 is a crucial component of senescent cell secretory phenotype and also the founding DAMP member (Davalos et al., 2013). In astrocytes, HMGB1 signaling has been reported to activate the secretion of a specific subset of inflammatory factors, e.g., matrix MMP-9, cyclo-oxygenase-2 (COX-2) and several chemokines that can facilitate monocyte infiltration (Pedrazzi et al., 2007). Moreover, astrocytes aged *in vitro* were shown to secret EVs that have a negative impact on the differentiation of oligodendrocytes (Willis et al., 2020). Interestingly, the pro-inflammatory phenotype of astrocytes in the aging brain can have several detrimental but also neuroprotective functions, proving that factors secreted by brain cells may modulate the microenvironment in apparently a more complex way than expected.

Besides astrocytes, also senescent microglia might influence the pro-inflammatory state in the brain. Experiments performed on microglia derived from rat brain cultured *in vitro* indicated that those cells can undergo stress-induced senescence, which is accompanied by SASP (Cao et al., 2020). Moreover, it was demonstrated in the elegant studies performed by Sierra et al. (2007) that microglia derived from old mice are characterized by higher granularity (flow cytometry analysis) and significantly increased expression of TNFα, IL-1β, IL-6. Altogether, this suggests that cytokines produced by aged microglia my represent SASP factors.

A recent study adds new insights into the connection among inflammation, brain function, DNA damage and telomeres. Namely reduction of Ft1 (a telomere-associated protein named AKTIP in humans) level in mice causes telomere aberrations, DNA instability and cell senescence. Ft1 mutant mice share similarities with lamin mutant animals, which are models for human progeroid syndromes, linking the Ft1 model to premature aging and progeroid diseases. The studies showed significant higher levels of both IL-6 and TGF-β in Ft1 knockout brains as compared to age matched control mice. Thus, it was concluded that the path to seizures generated following Ft1 reduction could start with DNA damage, including telomere dysfunction, followed by p53 activation, cell senescence, and SASP (Burla et al., 2018).

**Figure 4:**
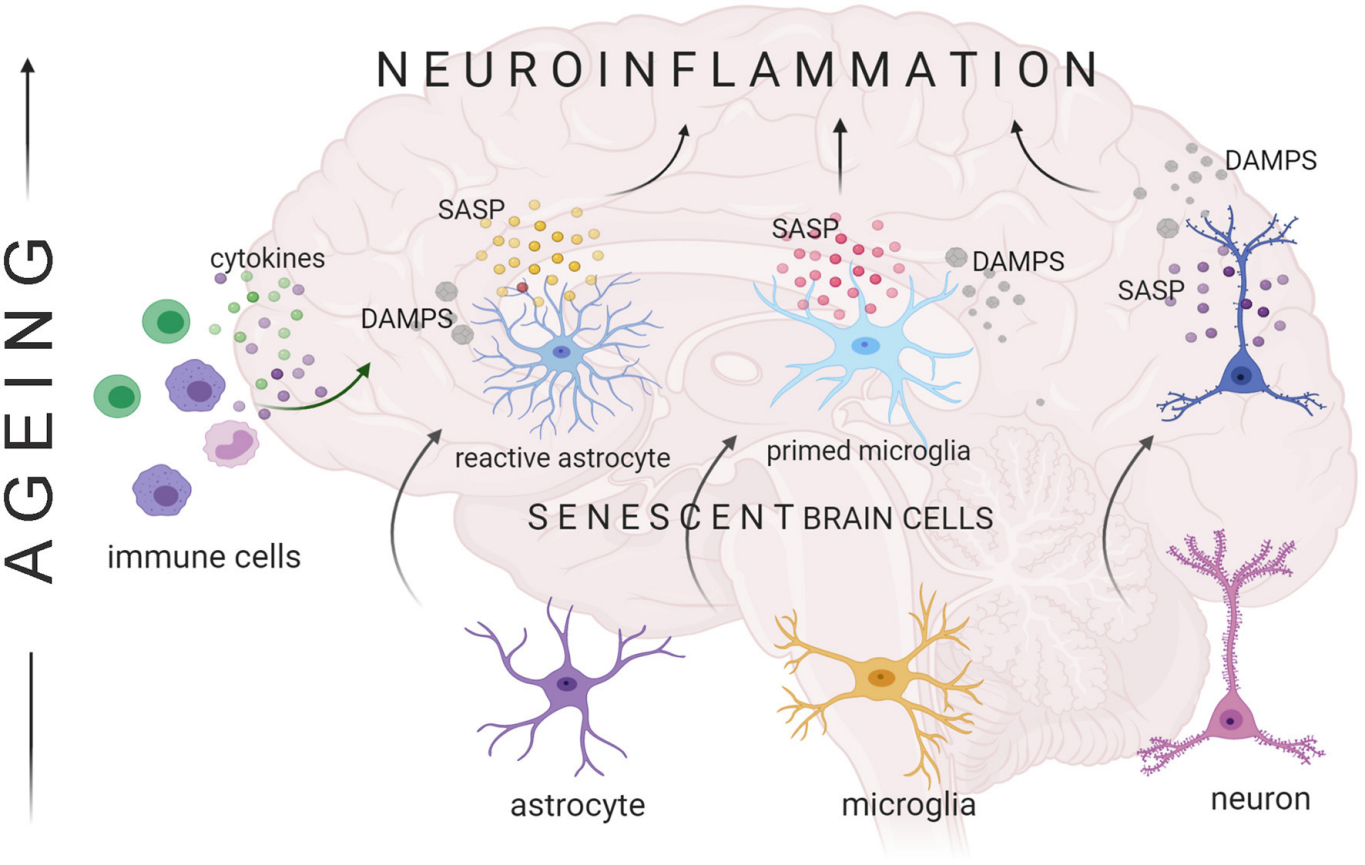
Senescent cells contribute to aging-related neuroinflammation. Glia and neurons can undergo cellular senescence, the characteristics of which includes SASP (senescence-associated secretory phenotype). Secretion of bio-active factors like cytokines, chemokines, extracellular matrix modifying enzymes etc. boosts the pro-inflammatory state observed in the aging brain. Release of senescent cell-derived cellular components that are recognized by neighboring cells as danger-associated molecular patterns (DAMPs) triggers inflammatory gene expression in brain cells.

Summarizing, there is an unquestionable and compelling evidence that, with age, there is a progressive development of a pro-inflammatory state of the brain. This increasing inflammation reflects both systemic and brain-derived changes of the immune cells but also results from the accumulation of senescent cells. Senescent brain cells secrete pro-inflammatory cytokines and represent a source of damaged macromolecules that generate the pro-inflammatory response in the brain microenvironment. Altogether, age-related changes in the cytokine milieu within the brain create harmful conditions that negatively influence CNS functioning (Figure 4).

## Senolytics in Brain Rejuvenation

As we get old, neuronal complexity declines. Dendritic arborization, length, synapse number, and spine density decrease to variable degrees in cortical areas. This is accompanied by reductions in most aspects of cognitive performance including memory, awareness, and intellectual abilities (Zhu et al., 2020). It can be speculated that brain aging, similarly to that of other organs, is caused by senescent cells. Senescent cell burden is low in young individuals, but increases with age in all tissues, especially in the brain, adipose tissue, skeletal muscle, kidney, skin, and ovaries (Sikora et al., 2010; Sikora, 2013). Recently, it has been shown that targeting senescent cells can alleviate the effect of brain aging, similarly to other organs, which gives the hope for increasing the cognitive performance and healthspan (Baker and Petersen, 2018; Sikora et al., 2019). The just emerging new therapeutic approach, senotherapy, that makes employs senolytics (the name originates from the words “senescence” and “lytic”), which are able to directly eliminate senescent cells, showed that, indeed, health improvement is within a therapeutic range (Baker and Petersen, 2018; Sikora et al., 2019; Kirkland and Tchkonia, 2020).

Senolytics turned out to be promising in preclinical studies of multiple conditions in mice, including metabolic disorders, cardiovascular disorders, cognitive impairment, pulmonary dysfunction, frailty, kidney and liver dysfunction, and osteoarthritis, but they also delayed cancer onset and extended the healthspan and lifespan (Tchkonia et al., 2020).

**Figure 5:**
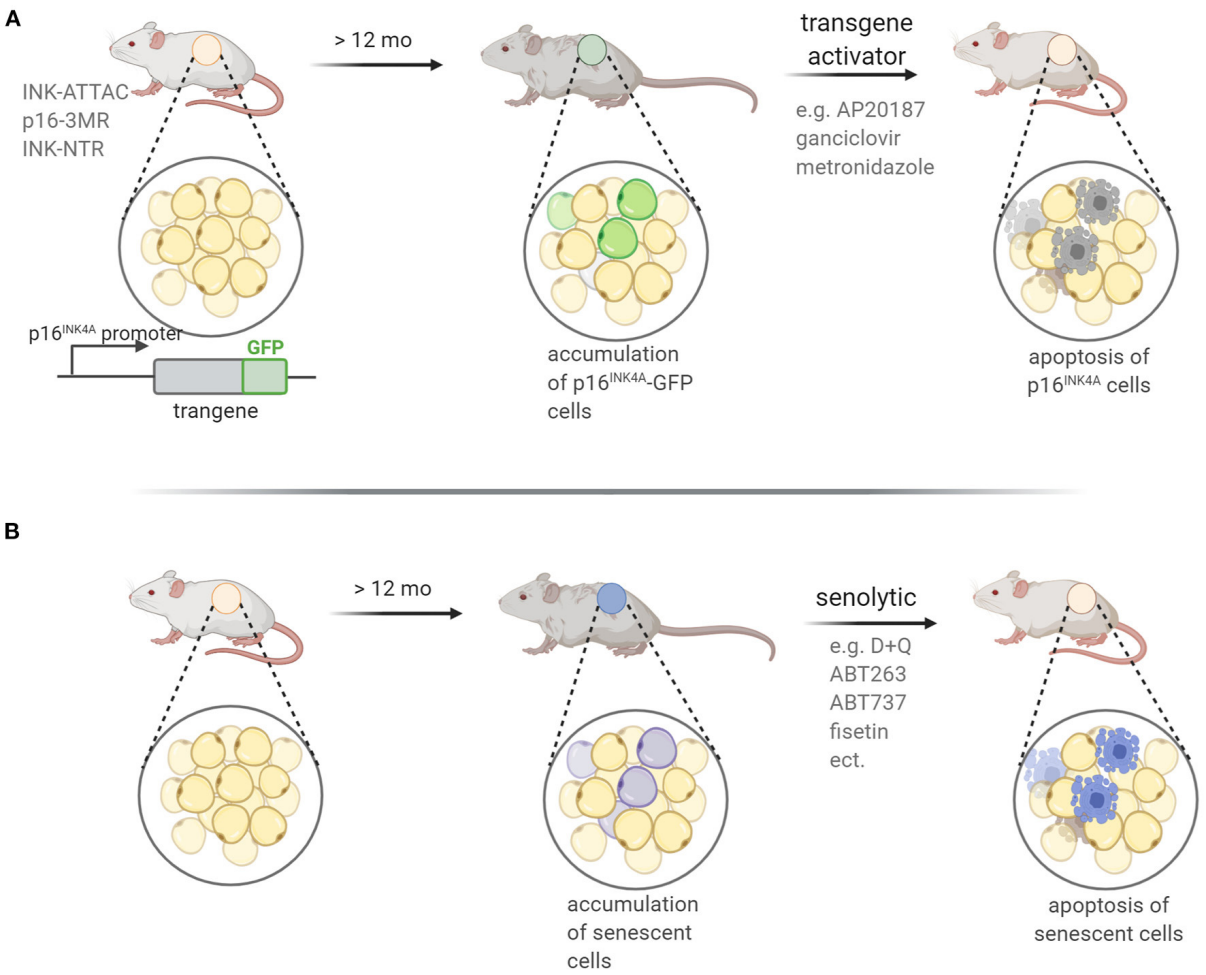
Mouse models applied in experimental senotherpy. Two experimental approaches for the eradication of senescent cells are used: **(A)** transgenic mouse in which transgene expression is under control of the promoter of a senescence-specific gene coding for p16^INK4A^ or p19^ARF^ protein. Activation of the transgene *via* an adequate compound leads to induction of cell death in p16^INK4A^ or p19^ARF^-expressing cells. Additionally, transgene might carry GFP protein sequence enabling identification of GFP-positive senescent cells before and after treatment; **(B)** senolytic treatment of wild type mice leads to death of senescent cells; death-inducing activity of senolytics stems from the ability to inhibit prosurvival or antiapoptotic pathways that are upregulated in senescent cells.

Several transgenic mouse models have been developed enabling the visualization, assessment and eradication of senescent cells *in vivo* (Figure 5A). In these models the promoter of the p16^INK4A^ gene was used to drive expression of genes encoding proteins, which induced death of senescent cells upon transgene activation. The same gene promoter was used to drive expression of fluorescent proteins in some of those mice. Using these animal models it was possible to confirm that senescent cells accumulate during aging and in response to environmental stresses, tissue damage or injury as well as at the sites of age-related pathologies. In INK-ATTAC animals, the p16^INK4A^ promoter drives the expression of an inducible caspase-like transgene, which encodes an apoptotic protein that is activated by a small ligand, AP20187 (Baker et al., 2011). Another model carrying suicide transgenes for a truncated herpes simplex virus thymidine kinase (p16-3MR mouse) under control of the *p16INK4A* promoter, have been developed. In this cases, selective killing of senescent cells was accomplished through treatment with ganciclovir (GCV) (Demaria et al., 2014). There is also a study, in which a p19ARF-directed *CDKN2A* gene promoter sequence that regulates expression of the diphtheria toxin receptor (ARF-DTR) was used (Hashimoto et al., 2016). Importantly, thanks to the transgenic mouse models that facilitate selective elimination of senescent cells, it has been shown that age-related pathological symptoms could be alleviated and, consequently, the animal healthspan and even the lifespan could be improved (reviewed in Sikora et al., 2019). On the other hand, transplantation of even a small number of senescent cells (preadipocytes) into young mice led to spreading of cellular senescence to neighboring tissues and caused persistent physical dysfunctions. This effect, along with lifespan reduction, could be counteracted by oral administration of senolytics (Xu et al., 2018).

The senolytic treatment, without genetic manipulations, has already been applied in many mouse models of age-related disease (Figure 5B). In general, senolytics can be classified into BCL family inhibitors, PI3K/AKT inhibitors, and FOXO regulators (Zhu et al., 2020). Briefly, the BCL family is composed of anti-apoptotic and pro-survival proteins, including BCL-2 and BCL-xL, the selective inhibition of which has been previously shown to cause cell death in some cancers (e.g., Souers et al., 2013). Presently, a similar strategy has been adopted to effectively remove senescent cells (Short et al., 2019). At present, BCL inhibitors with senolytic effects include navitoclax (namely ABT263), A1331852, A1155463, and ABT737. Another pro-survival pathway active in senescent cells is the PI3K/AKT pathway, which can be targeted by dasatinib and quercetin. Dasatinib (D), an anticancer drug, is a tyrosine kinase inhibitor that can affect a variety of tyrosine kinases (Montero et al., 2011). Quercetin (Q), is a natural flavonol that inhibits the activity of mTOR and PI3K (Bruning, 2013). D and Q are the first senolytic drugs to be discovered *via* a hypothesis-driven approach, and they have been demonstrated (used in combination, D+Q) to alleviate symptoms of a variety of age-related diseases in mouse models and to improve survival in older mice (Kirkland and Tchkonia, 2020). The D+Q combination has been tested in clinical trials and the results show that D+Q significantly improve the physiological functions in idiopathic pulmonary fibrosis (IPF) patients (Justice et al., 2019) and can effectively eliminate p16*INK4a*-positive cells, reduce the activity of SA-β-gal, and attenuate the release of inflammatory factors in patients with diabetic nephropathy (Hickson et al., 2019). Epidemiological studies have suggested that flavonoid intake has beneficial effects on vascular health, and is associated with a decreased risk of coronary heart disease and cardiovascular disease. Fisetin is a natural flavonoid found in many fruits and vegetables, such as apples, persimmons, grapes, onions, cucumbers, and strawberries (Khan et al., 2013). In the nervous system, fisetin could inhibit the activity of lipoxygenase and reduce the production of pro-inflammatory eicosanoids and their by-products, and thus protect brain function in age-related neurological diseases (Maher, 2012). Fisetin treatment reduced the burden of senescent cells, inflammation, and oxidative stress in premature aging mice, while in elderly mice it could restore tissue homeostasis, reduce age-related pathological changes, and extend the median and maximum lifespan (Yousefzadeh et al., 2018).

Another target of senolitics is HSP90. HSP90 is a highly conserved chaperone protein that plays an important role in protein stabilization and degradation. A previous study has shown that 17-DMAG inhibits HSP90, downregulates the PI3K/AKT pathway, reduces the number of senescent cells, and promotes senescent cell apoptosis. Treatment of Ercc1–/Δ mice with 17-DMAG can significantly lower the incidence of age-related dysfunctions (Fuhrmann-Stroissnigg et al., 2017). Other examples are FOXO regulators—for example a peptide named FOXO4-DRI, which binds p53 with high affinity. Disrupting the p53-FOXO4 interaction using FOXO4-DRI caused p53 to be excluded from nucleus and directed to mitochondria for induction of apoptosis, ultimately eliminating the senescent cells. FOXO4-DRI has been shown to restore fitness, fur density, and renal function in both rapidly aging Xpd^TTD^/TTD mice, and naturally aging mice (Baar et al., 2017). Recently, it has been shown that FOXO4-DRI selectively induced p53 nuclear exclusion and apoptosis in senescent Leydig cells. In naturally aged mice, FOXO4-DRI improved the testicular microenvironment and alleviated age-related testosterone secretion insufficiency (Zhang et al., 2020).

Although data from preclinical studies, showing the positive effect of senolytics on reducing the symptoms of aging-related diseases, are already very abundant, research on their beneficial effects on the nervous system is still scarce.

Senescence-accelerated prone 8 (SAMP8) mouse is one of the models of aging that exhibits a progressive, age-associated decline in brain function similar to human AD patients. As they age, SAMP8 mice present with an early deterioration in learning and memory and a number of pathophysiological alterations in the brain, including increased oxidative stress, inflammation, vascular impairment, gliosis, Aβ accumulation, and tau hyperphosphorylation (Cheng et al., 2014). Young SAMP8 mice were fed with fisetin for 7 months. The effects of the fisetin-enriched diet was assessed in old SAMP8 mice and the development of age-related changes were compared to the young SAMP8 animals. As it was observed in Barens maze and open field test, fisetin prevented cognitive and locomotor deficits that develop with age in SAMP8 mice. Dysregulation of neuronal homeostasis and stress responses was partially prevented and markers of increased inflammation were partially reduced in the hippocampus of old SAMP8 mice receiving fisetin. However, the study did not provide evidence of direct elimination of senescent brain cells by fisetin.

Recently, Chinta et al. (2018) have shown that in senescent astrocytes *Cdkn2a* mRNA and SASP factors (IL-6, IL-1α, IL-8, and MMP-3) were elevated in substantia nigra of paraquat-induced PD p16-3MR mouse model. This was correlated with the loss of DA (dopaminergic) neurons, motor dysfunction, and reduced neurogenesis. Injection of ganciclovir, which facilitated selective depletion of senescent cells, improved motor function and restored adult neurogenesis in these mice (Chinta et al., 2018). Another study revealed that there is accumulation of p16^INK4A^-positive senescent astrocytes and microglia in a mouse model of tau-dependent neurodegenerative disease. Clearance of these cells, as they arise in INK-ATTAC transgenic mice, prevents gliosis and hyperphosphorylation of both soluble and insoluble tau, thus preserving cognitive function. Pharmacological intervention with the ABT-263 senolytic resulted in similar effects as genetic intervention (Bussian et al., 2018). Subsequently Zhang et al. (2019) proved that D+Q was able to reduce neuroinflammation, lessen Aβ load and ameliorate cognitive deficits in the familial mouse model of AD, that is, APP/PS1 AD mice. Interestingly, this was possible by selective removal of senescent oligodendrocytes from the plaque environment.

Senescent glial cells can also affect other, namely neuropsychiatric, functions of the brain. To investigate the role of senescence in obesity-related neuropsychiatric dysfunction, Ogrodnik et al. (2019) used the INK-ATTAC mouse model, from which p16^INK4A^-expressing senescent cells can be eliminated. They found that obesity in mice led to accumulation of senescent glial cells in the proximity of the lateral ventricle, a region in which adult neurogenesis occurs. Clearing senescent cells from leptin receptor-deficient obese mice, or mice fed with high fat diet, by treatment with D+Q restored neurogenesis and alleviated anxiety-related behavior. This study proved that senescent glial cells are major contributors to obesity-induced anxiety and that senolytics represent a potential new therapeutic avenue for treating neuropsychiatric disorders.

The so far mentioned reports concern beneficial effects of eradication of senescent non-neuronal cells on neurological disorders. However, very recently it has been shown that both approaches, namely in genetically modified INK-ATTAC mice and pharmacologically treated mice resulted in senescent microglia eradication and cognitive improvement in healthy mice (Ogrodnik et al., 2021).

To our knowledge there are also two published papers reporting on senolytic-driven elimination of neuronal cells. One concerns chemotherapy-induced peripheral neuropathy and another taupathy in mouse models (Musi et al., 2018; Acklin et al., 2020).

Chemotherapy-induced peripheral neuropathy is among the most common dose-limiting adverse effects of cancer treatment. Recently, it has been shown that a commonly used anticancer drug, cisplatin, caused accumulation of senescent neuronal cells in dorsal root ganglia (DRG). Drug treatment was associated with cisplatin-induced peripheral neuropathy (CIPN) in mice. The features of senescence in DRG included: SA-β-gal increased activity, accumulation of cytosolic p16^INK4A^ and HMGB1, and increased expression of p16^INK4A^, p21, and MMP-9. Both the pharmacological treatment with the ABT263 senolytic and utilization of genetically modified mice (p16-3MR mice) resulted in induction of apoptosis of p16^INK4A^-positive cells. In consequence, clearance of senescent neuronal cells and reversed of cisplatin-induced peripheral neuropathy in mice were observed (Acklin et al., 2020).

The accumulation of tau protein is the most common pathology of AD (Orr et al., 2017). Tau-containing neurofibrillary tangles (NFTs) correlate with disease severity in human AD (Arriagada et al., 1992). NFT-containing neurons are long-lived and not prone to immediate cell death (de Calignon et al., 2009). Interestingly, Musi et al. found that in a mice model of taupathy and postmortem human brain tissue NFT-containing neurons have upregulated expression of genes involved in cell survival and viability, inflammation, cell cycle and molecular transport and downregulated expression of genes involved in apoptosis, necrosis, and cell death pathways (Musi et al., 2018). TauNFT mouse brains displayed significantly elevated histone γH2AX level, and several- fold higher *Cdkn1a* (p21^CIP/WAF^) and *Cdkn2a* (p16^INK4A^) and SASP gene (*Il1b, Cxcl1, Tnfa, Tlr4*) expression than control mice, suggesting that NFT-containing neurons are senescent. Intermittent D+Q treatment significantly reduced the number of NFT-containing cortical neurons and NFT associated senescence gene expression was reduced by D+Q. Moreover, D+Q-treated mice expressed significantly higher levels of neuronal proteins (NeuN, PSD95), but the astrocyte protein GFAP level was unchanged, while microglia *Iba1* expression was elevated. This suggests that D+Q-mediated neuroprotection and decreased SASP were not due to reduction in pro-inflammatory glia (astrocytes or microglia) but, instead, could be associated with fewer NFT-containing neurons. Moreover, these data suggest that removal of these neurons with D+Q produced long-lasting global effects on brain, as evidenced by histopathology and MRI analyses.

## Discussion

Aging affects all organs and tissues in our body, but aging-related changes in the brain seem to be particularly devastating as they directly, negatively, influence our memory, cognitive ability and intellect, and indirectly limit our physical ability. All of this together leads to reduction in the quality of life in its last decades. Due to civilization development and progress in science, medicine and technology, human life expectancy has increased significantly in the last century, thus, the quality of life in old age is of particular importance. For many years biogerontology has been involved in revealing biological mechanisms of the aging process, however, just recently, researchers became aware that age-related disease mechanisms should be found at the roots of the biology of aging (Kennedy et al., 2014). This also concerns the brain. At the roots of neurodegenerative disorders there are the same molecular and cellular mechanisms that are responsible for “normal” aging. The difference is in the intensity of these processes and the chronology of their appearance. That is why it is so important to answer the question how the brain without symptoms of neurodegeneration is aging? The answer to this question is as complex as the brain but is necessary to open the avenue for treatment of age-related cognitive decline.

Astonishingly, despite the unique morphological and cellular complexity of the central nerve system, many hallmarks typical for senescence identified in other tissues have been found also in the brain (Mattson and Arumugam, 2018). Some of them, such as low grade inflammation, epigenetic and translational changes, proteostasis and autophagy disturbances, seem to be common for aged brain and other tissue senescence. Thus, it is reasonable to hypothesize that cellular senescence may give the ground for aging-related changes that significantly alter brain function. Accordingly, it has been noticed recently that cellular senescence can play a pivotal role in brain aging (Tan et al., 2014; Baker and Petersen, 2018) and elimination of senescent cells by senolytics can alleviate neurodegeneration symptoms in mice models (reviewed by Sikora et al., 2019). The following questions arises: which brain cells are the main target of senotheraphy and what will be the effect of eradication of senescent cells in aged brain that cannot be effectively replaced by new ones like, for example, neurons? Even more, if regenerated to a certain extent, can the functionality of the neuronal network be restored afterwards? So far, the beneficial effect of senotherapy in mice models of neurological disorders was improvement in health due to eradication of senescent glia (Bussian et al., 2018; Ogrodnik et al., 2019) or oligodendrocytes (Zhang et al., 2019). However, two recent papers reported also reduction in the number of senescent nerve cells (Musi et al., 2018; Acklin et al., 2020). It suggests that senescent glia cells represent a population of brain proliferation-competent cells that become sensitive to senolytic treatment due to expression of common markers of senescence. On the contrary, senescent neurons, as non-dividing post- mitotic cells, acquire a specific senescent phenotype that could be not easily targeted by senolytic agents. Still the question is whether elimination of senescent brain cells can postpone aging and age-related cognitive impairment or if it is only restricted to pathological conditions associated with increased accumulation of senescent cells? Further studies are needed.

Although much attention is focused on the anti-aging strategy based on senolytic therapy approach, senomorphic agents, that modulate the senescent phenotype (Childs et al., 2017) might also be considered particularly useful in postponing brain aging. This supposition stems from strong experimental support showing that both autophagy and inflammation, strictly connected with senescence, have profound impact on synaptic plasticity. Accordingly, one of the theories of age-dependent synaptic dysfunction leading to learning impairments, focuses on failing autophagy in presynaptic terminals. Distal axons and presynaptic terminals are widely considered the primary site of autophagosome biosynthesis. With aging autophagosome formation is impaired, which makes neuronal autophagy dysfunctional. In line with this, it has been shown that autophagosome biogenesis decreases with age (Stavoe et al., 2019). Additionally aged-dependent increase of damaged lysosomes was found leading to autophagy inhibition at the degradation stage (Gomez-Sintes et al., 2016; Stoka et al., 2016). The turnover of presynaptic proteins, taking place in so called active zones (specific presynaptic structures specialized in neurotransmitter release), was shown to be altered with aging, causing upscaling of the active zone size and abnormal release of synaptic vesicles (Maglione et al., 2019). At some point, synapses may reach their upper limit of plasticity potential and lose ability to change, which may translate into learning deficits (Bhukel et al., 2017). Moreover, since defective mitochondria are being sequestrated by autophagosomes, aging may lead to dysfunctional mitophagy and increased oxidative stress. Increased oxidative stress, in turn, leads to LMP that contribute to further impairment of autophagy and accumulation of damaged proteins and organelles. This vicious cycle may lead to an age-related decrease in the number of mitochondria in synaptic terminals and to functional impairments (Ojo et al., 2013; Rango and Bresolin, 2018). Mitochondria also play a key role in Ca^2+^ homeostasis. A drop in the numbers of mitochondria decreases the ability of the synapse to regulate Ca^2+^ level, which may lead to synaptic dysfunction or even cell death because mitochondrial Ca^2+^ overload induces pro-apoptotic pathways (Devine and Kittler, 2018). Recently, non-canonical role of autophagy machinery on microtubule-dependent axonal transport was found (Negrete-Hurtado et al., 2020). Thus, autophagy activation promote not only degradation but also may ameliorate axonal transport. Interestingly, spermidine, a polyamine compound, the level of which decreases with age, has been shown to have significant anti-aging potential, including beneficial effects on cognitive performance in elderly people at risk of dementia (Wirth et al., 2018). Its beneficial action on the cognitive performance may be associated with promotion of autophagy. It has been shown that simply feeding aged mice with spermidine partially reversed age-related deficits in LTP at mossy fiber-CA3 synapses in the hippocampus (Maglione et al., 2019). In line with those results, other modulators of autophagy alleviate age-related cognitive deficits in rodents (Glatigny et al., 2019). Thus, just by regulating one of the effector mechanism of cellular senescence—autophagy, an improvement in cognition and memory might be expected without urgent need for elimination of senescent cells. Not only pharmacological treatment may cause upregulation of autophagy. Also physical exercises and diet were found to have a positive effect on both autophagy and the brain function (Andreotti et al., 2020).

Apart from autophagy, proinflammatory cytokines, which create a unique secretory phenotype of senescent cells, are undoubtedly the most obvious goal for efficient therapy directed against aged brain. There is great body of evidence that increased level of inflammatory cytokines has a deleterious effect on synaptic plasticity. It was demonstrated that upon stressful conditions or aging, the level of IL-1β increases leading to impairment of LTP in the hippocampus (Murray and Lynch, 1998). Accordingly, administration of IL-1β in supraphysiological concentration following learning, provided the first evidence that excess of this cytokine results in impaired memory. Importantly, low, physiological level of IL-1β improved memory (Goshen et al., 2007). Moreover, gene polymorphism analysis performed on over 5000 individuals under the frame of the PROSPER study revealed that a certain genetic variation in the interleukin-1β-converting enzyme (ICE) gene is associated with better performance in cognitive function tests and lower IL-1β production (Trompet et al., 2008). Similarly to IL-1β, also TNFα, at levels over the physiological range, leads to hippocampal LTP impairment associated with depressive-like behavior and cognitive deficits in animal models (Butler et al., 2004). Other studies have revealed IL-6 as another example of SASP component that significantly influences brain function. When IL-6 is elevated in the brain of mice they display impaired cognitive abilities, deficits in learning, abnormal anxiety traits and habituations, and decreased social interactions concomitantly with significant disruption of synaptic transmission and structural plasticity (Wei et al., 2015). Thus, age-associated supra-physiological levels of proinflammatory cytokines should contribute to defects in brain functionality. Indeed recent studies revealed that anti-inflammatory agents, such as ibuprofen, protect from cognitive decline induced by chronic inflammation in genetic mouse models by decreasing markers of senescence in neurons (Fielder et al., 2020).

**Figure 6:**
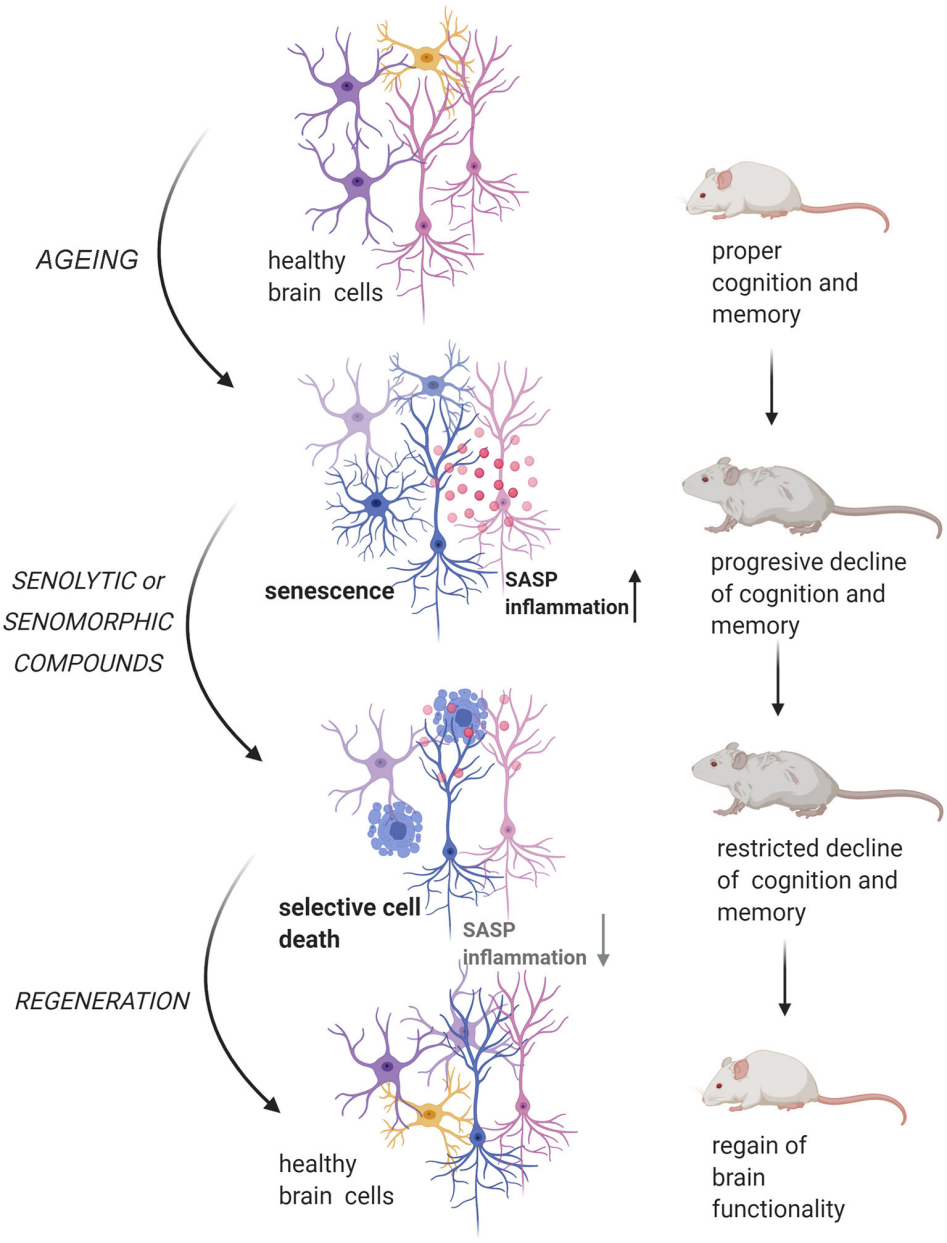
The potential role of senotherapy in counteracting aging-related brain dysfunction. The cell-autonomous and non-autonomous features that accompany senescence of brain cells may lead to cognition impairment and memory decline. Treatment of aged organism with senolytic or senomorphic compounds entails the elimination of senescent cells or, alternatively, lower the deleterious influence of senescent cells on brain microenvironment (SASP reduction). In consequence, restoration of proper brain activity may be achieved.

Although age-related changes in the brain have been widely recognized for many years, only recently we are witnessing accumulation of experimental proof of the cellular bases of those changes. Moreover, therapeutical approaches that rely on pharmacological elimination or modification of senescent cells give hope for efficient restraining of age-related disease, including neurodegeneration (Figure 6). However, the open question still remains weather we will be able to restrict the accumulation of progressive changes in the brain that appear while we age? Can we safely intervene into the CNS without influencing its proper functioning? So far collecting data, including the very recent one showing that both genetic modification (ATTAC mice) and pharmacological intervention (D+Q) significantly improved cognitive function in aged mice (Ogrodnik et al., 2021) give as the hope for more satisfactory late years of our life, even if some clinical trials are required. The problem is that the process of “normal” aging is still not fully accepted as a subject of clinical trials. However, the first ongoing clinical trial with using metformin as an antiaging agent (https://www.longevity.technology/worlds-first-anti-aging-trial-gets-green-light/) paved the way to other approaches aimed at pharmacologically targeting the aging process in order to preserve cognitive ability.

## Author Contributions

ES made substantial contributions to the conception and design of the review and gave final approval of the version to be published, ES, AB-Z, MD, GM, AK, MW, and JW participated in writing the particular chapters of the manuscript and approved the final version. GM, AK, and MD prepared figures. All authors contributed to the article and approved the submitted version.

## Conflict of Interest

The authors declare that the research was conducted in the absence of any commercial or financial relationships that could be construed as a potential conflict of interest.
